# Cracking the code of neuronal apoptosis and survival

**DOI:** 10.1038/cddis.2015.309

**Published:** 2015-11-05

**Authors:** S Cavallaro

**Affiliations:** 1 Department of Biomedical Sciences, Institute of Neurological Sciences, Italian National Research Council, Via Paolo Gaifami 18, 95126 Catania, Italy

## Abstract

Neuronal apoptosis and survival are tightly controlled processes that regulate cell fate during the development of the central nervous system and its homeostasis throughout adulthood. A new study in primary cultures of cerebellar granule neurons identified common transcriptional cascades during rescue from apoptosis by insulin-like growth factor-1 (Igf1) and pituitary adenylyl cyclase-activating polypeptide (Pacap), thus suggesting the existence of a high degree of conservation of cell survival pathways.

Neuronal apoptosis represents an intrinsic suicide program by which a neuron orchestrates its own destruction. During normal nervous system development, physiologically appropriate neuronal loss contributes to a sculpting process that removes approximately one-half of all neurons born during neurogenesis. Neuronal loss subsequent to this developmental window is physiologically inappropriate and can contribute to neurodegenerative disorders such as Alzheimer's and Parkinson's disease. Hence, elucidating the molecular mechanisms underlying neuronal apoptosis may contribute to understanding the basis of human neuropathology and help to identify novel treatment and prevention strategies.

Cerebellar granule neurons (CGNs) are the most abundant neuronal cell type in the mammalian brain and represent, either *in vivo* or *in vitro*, the most common used model to study neuronal apoptosis. Primary cultures of CGNs, in particular, have been extensively utilized to examine the signal transduction mechanisms underlying neuronal apoptosis. In this *in vitro* paradigm, CGNs undergo rapid and synchronous apoptotic cell death within 24 h after removal of serum and lowering of extracellular potassium.^1^ This form of cell death requires protein transcription and synthesis, becoming irreversible after the first 6 h following its induction.^1^ Before this ‘commitment point' CGNs can be rescued by the activation of specific signal transduction pathways or by the treatment with specific neurotrophic factors, such as Igf1^1^ and Pacap.^2^ We and others have spent the last 20 years searching for the signaling pathways underlying neuronal survival by these growth factors. Although their effects are mediated by different receptors and intracellular second messengers,^2–7^ their signaling pathways converge into the nucleus and regulate gene expression.^6, 8–10^

In recent years, the advent of high-throughput technologies has offered a new approach to decipher the transcriptional mechanisms underlying neuronal apoptosis and survival, unraveling a systems biology based perspective.^11^ Indeed, the ability of a neuron to promote or evade apoptosis depends on the activity of an integrated network of genes and their encoded proteins, which never work alone but interact with each other in highly structured and incredibly complex ways, forming molecular circuits that correspond perfectly to cell functional specifications.

By using whole-genome expression profiling, the transcriptional mechanisms controlling the apoptotic/survival switch in CGNs have now been investigated. Commitment to apoptosis and rescue by Igf1 or Pacap produce a large impact on the CGNs transcriptome, regulating the expression of specific genes and functional pathways.^12^ Surprisingly, the survival effects of Igf1 and Pacap share striking similarities and are propagated by common transcriptional cascades (Figure 1).

This is the first time neuronal apoptosis and survival cross paths are investigated, and that the transcriptional effects of two rescue factors are compared. Although these findings represent the first glimpse into the transcriptional landscape of neuronal apoptosis and survival, the convergence of Igf1- and Pacap-induced effects suggests the existence of a conserved transcriptional program underling neuronal survival. Interestingly, several proteins encoded by differentially expressed genes, at the intersection of apoptosis and survival, are targets of pharmacological compounds. Their exploitation, therefore, may help interfering with the intracellular pathways involved and guide novel therapeutic strategies for neurodegenerative diseases.

Further studies are still required to analyze the time-dependent dynamics of gene regulation during the induction of apoptosis and the rescue by trophic factors. Additional studies are also needed to investigate how universal is the transcriptional program governing apoptosis and survival in different neurons, and also in other cell types and species. The evolutionary course of fundamental transcriptional processes, such as those controlling cell life and death, could have been carried out under rigid constraints. Thus, it may not be surprising if their transcriptional mechanisms are conserved across cells and species, and could be activated by redundant signals like those induced by Igf1 and Pacap in CGNs following induction of apoptosis.

Until now we have focused our attention on the hardware, the next challenge will be to uncover the ‘regulatory software' controlling neuronal fate. Cracking the code of apoptosis and survival is complex but nowadays feasible. It will require a multidisciplinary team of molecular biologists, mathematicians, computer scientists and systems biology engineers. There will come a time when neuronal life or death will be seen as the result of a software-controlled gene/protein network, rather than the expression or activity of a single gene/protein. Then, we will be able to interpret neurodegeneration not only as a variation in gene or protein (hardware) segments, but also in regulatory (software) elements. This new perspective will allow us to implement an innovative pharmacology based on systems biology and focused on downstream targets or networks, rather than membrane receptors interference, as we have done until today. Similar to computers, where the most common problems arise from buggy software, our neurons may probably deal with frequent mulfunctions in transcription software. Thus, cracking the code of neuronal life or death will allow us to reach a higher level analysis and, hopefully find a patch for neurodegeneration.

## Figures and Tables

**Figure 1:**
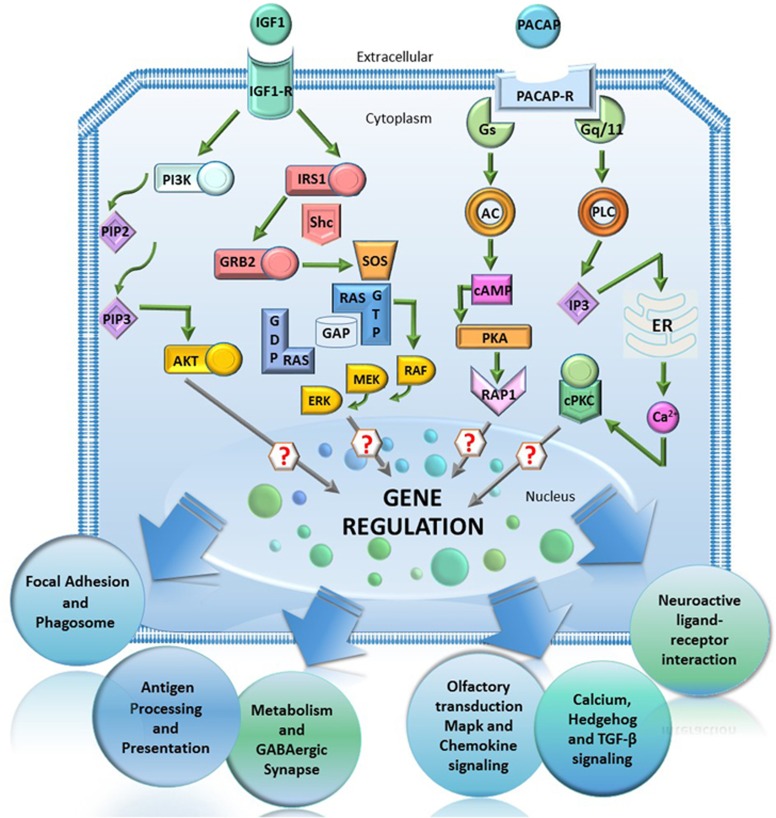
The survival effects of Igf1 and Pacap are propagated by common transcriptional cascades. Primary cultures of cerebellar granule neurons can be rescued from apoptosis by the treatment with Igf1 and Pacap. Although depending on different upstream signaling pathways, their survival effects converge into common transcriptional cascades, thus suggesting the activation of a conserved transcriptional program underlying neuronal survival
